# Evaluation of Void Defects behind Tunnel Lining through GPR forward Simulation

**DOI:** 10.3390/s22249702

**Published:** 2022-12-11

**Authors:** Xianlong Wu, Xiaohua Bao, Jun Shen, Xiangsheng Chen, Hongzhi Cui

**Affiliations:** 1 College of Civil and Transportation Engineering, Shenzhen University, Shenzhen 518060, China; 2 Key Laboratory for Resilient Infrastructures of Coastal Cities MOE, Shenzhen University, Shenzhen 518060, China

**Keywords:** tunnel lining, ground-penetrating radar (GPR), finite difference time domain (FDTD), void, forward modeling

## Abstract

Voids, a common defect in tunnel construction, lead to the deterioration of the lining structure and reduce the safety of tunnels. In this study, ground-penetrating radar (GPR) was used in tunnel lining void detection. Based on the finite difference time domain (FDTD) method, a forward model was established to simulate the process of tunnel lining void detection. The area of the forward image and the actual void area was analyzed based on the binarization method. Both the plain concrete and reinforced concrete lining with various sizes of air-filled and water-filled voids were considered. The rationality of the model was verified by measured data. It was observed that the response mode of voids can be hyperbolic, bowl-shaped, and strip-shaped, and this depends on the void’s width. Compared with the air-filled voids, water filling increases the response range of the voids and produces a virtual image. Although the diffracted wave caused by a steel bar will bring about significant interference to the void response, the center position of the voids can be accurately located using 3D GPR.

## 1. Introduction

Owing to the influence of the geological environment and the limitations of construction technology, there are many types of defects in tunnel engineering, among which voids are the most common construction defects. The void is the weak point of the tunnel structure and under the action of ground stress or traffic load, these weak points will become the starting point of tunnel structure quality deterioration, leading to lining cracking, water leakage, and other problems, reducing the safety of the tunnel [1]. Therefore, it is necessary to detect the tunnel lining, find the void, and repair it as soon as possible to ensure the safety of the tunnel.

Ground penetrating radar (GPR) is a technology that uses high-frequency radio electromagnetic waves to perform non-destructive testing of targets in the medium [2,3,4]. Owing to its advantages of high resolution, high efficiency, intuitive results, and non-destructive nature, it is widely used in geological exploration [5], road surface inspection [6], underground pipeline detection [7], archaeological detection [8,9], etc. In addition, GPR is also used in the detection of tunnel linings [10,11]. These tunnel applications include lining damage assessment (lining cracking, deformation), construction quality evaluation (steel bar quantity, protective layer), and lining void detection [12,13,14]. However, owing to the complexity of the tunnel environment, the void response is not easy to distinguish, and the recognition of the void greatly depends on the professional knowledge and work experience of the technicians. Therefore, establishing a standard method for void identification, determining the characteristics of void response under different conditions, and identifying the void objectively and accurately is an urgent problem to be solved.

Owing to the complex conditions of the tunnel site, it is impossible to accurately control all the variables; therefore, research on void detection is mainly performed through modeling tests and numerical simulations. Currently, with the improvement in computing power, numerical simulation technology has been able to accurately model the actual tunnel conditions and simulate the reflection, refraction, and scattering of electromagnetic waves in the medium. As a result, many methods for the forward modeling of electromagnetic waves, such as the method of motion (MoM), the finite element method (FEM), and the finite difference time domain method (FDTD) [15] have been widely studied. The FDTD method, first proposed by Yee [16], uses the discretization of time and space to solve the Maxwell equations. This method has been adopted by many scholars due to its high precision and efficiency [17,18,19,20,21]. Lin et al. [22] performed forward modeling of common lining defects and provided a standard for the interpretation of defect images. Lv et al. [23] studied the void electromagnetic field and developed a void imaging method. The research on GPR detection images is mostly limited to the influence of a single factor, and the studies of void response are mainly focused on the analysis of corresponding features. However, the relationship between the response characteristics and the actual void size is rarely investigated.

In this study, gprMax based on FDTD was used to establish the forward modelings considering the variables that may be encountered in actual engineering, such as void size, void position, lining type, and water filling. Before conducting the forward simulation, the electrical parameters of each part were determined by theoretical analysis and image comparison. Then, 2D models were used to analyze the influence of variables on the mode and extension area of the response. The relationship between the void size and response mode was analyzed, and the feasibility of estimating the void area by ground penetrating radar image was discussed. Next, the void detection process of 3D GPR was modeled forward using 3D models. The response characteristics of different measuring lines were analyzed, and the reference that can be used to locate the void position using 3D GPR was explained. This study aims to comprehensively analyze the influence of different variables on the void response characteristics, and provide a reference for tunnel structure defect prevention and safety maintenance.

The main contribution of this study is described as follows:

- Establishing a GPR forward model and analyzing the variables (width, thickness, inflation, and water filling) affecting the void response mode.
- Calculating the response area of the voids by binarization method and confirming the applicability of GPR to estimate the void area.
- Providing a method of determining the center of voids location according to the response intensity.

**Figure 1:**
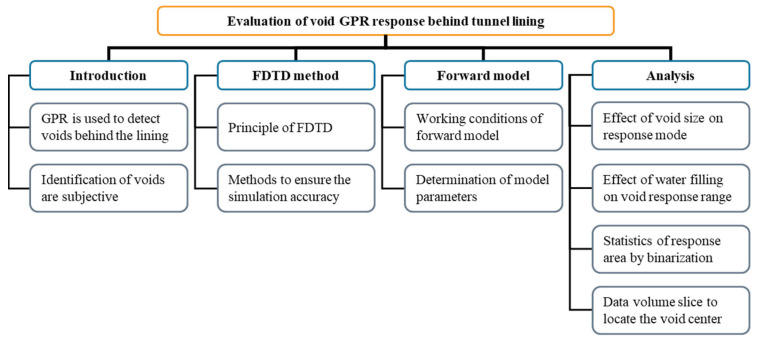
The research flowchart of this study including the research contents and methods.

The rest of this paper is organized as follows: In Section 2, the theoretical background of the FDTD and the methods to ensure the simulation accuracy are introduced. In Section 3, the working conditions of forward simulation are explained and the model parameters are determined. In Section 4, the influence factors on the response mode are discussed, and the advantages of 3D GPR for void detection behind tunnel lining are analyzed. Finally, the research work is summarized and discussed, and the shortcomings of this study and the following research plan are explained. The research flowchart of this study is shown in Figure 1.

## 2. Theoretical Background

GPR locates the target by using the reflection and scattering of modulated high-frequency electromagnetic waves in heterogeneous bodies [24,25]. Electrical parameters, such as the dielectric constant, conductivity, and permeability of the medium, have a profound impact on the final image. According to the electromagnetic wave propagation theory, the electromagnetic phenomena existing at the macro scale can be described by a set of Maxwell equations, which are used to represent the relationship between the basic electromagnetic field and their emission source, that is, the electric field. The expression of the first-order partial differential is Equations (1)–(4).

(1) $$\nabla \times E=-\frac{\partial B}{\partial t}$$

(2) $$\nabla \times H=\frac{\partial D}{\partial t}+J$$

(3) ∇⋅B=0

(4) ∇⋅D=ρ

where ***E*** is the magnetic field intensity; ***H*** is the electric field intensity; ***B*** is the magnetic induction intensity; ***D*** is the potential displacement vector; *t* is the time; ρ is the unit charge density; ***J*** is the current density; and ∇ is the Hamiltonian operator.

**Figure 2:**
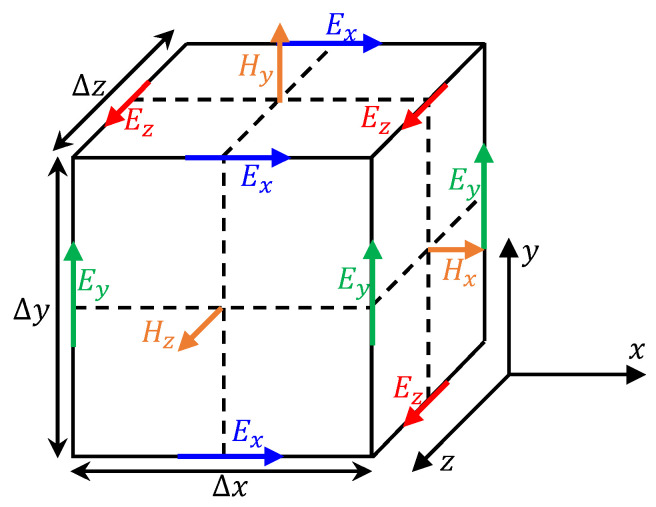
Yee grid of FDTD algorithm, E and H are electric field and magnetic field components, respectively. This kind of grid is used to discretize both continuous space and time; therefore, the propagation of electromagnetic waves in the medium can follow Maxwell’s law.

In the Maxwell equations, the field parameters are assumed to be functions of the time and space coordinates. Therefore, in order to realize the forward simulation of GPR and solve the Maxwell equation, it is necessary to determine the model geometry, boundary conditions (electromagnetic wave reflection or absorption), and initial conditions (electromagnetic wave source, frequency, and dipole distance). At the same time, electromagnetic waves should be allowed to propagate freely in the medium. In order to solve these problems, Yee [16] proposed the finite difference time domain (FDTD) method; the essence of this method is the discretization of the continuous space and time through the grid element (Figure 2) and the basic time increment. In addition, the partial differential form of the Maxwell equation was applied to each element. It can be seen that when the FDTD method is used to solve the Maxwell equation, the 3D size of the element mesh (Δ*x*, Δ*y*, Δ*z*) greatly affects the accuracy of the forward modeling. In general, considering the calculation error caused by the dispersion of electromagnetic waves after discretization, better accuracy can be obtained when the size of the discrete grid is less than 1/10 of the minimum wavelength, as shown in Equation (5).

(5) $$\Delta x=\Delta y=\Delta z<\frac{\lambda }{10}$$

where Δ*x*, Δ*y*, Δ*z* are the 3D size of the element mesh, and λ is the minimum wavelength.

Moreover, the time length Δ*t* of each iterative step in solving the equation also affects the accuracy of the forward results; the relationship between the time of the iterative step and the 3D size of the element grid can be expressed by Equation (6).

(6) $$\Delta t\leq \frac{1}{c\sqrt{\Delta x^{-2}+\Delta y^{-2}+\Delta z^{-2}}}$$

where c is the speed of electromagnetic waves in a vacuum, $2.99\times 10^{8}$ m/s.

## 3. Forward Simulation Based on FDTD

In this section, gprMax based on FDTD is used to simulate the process of GPR lining detection. This software is widely used to simulate the propagation law of electromagnetic waves in isotropic homogeneous bodies [26,27,28,29,30]. To save calculation time and facilitate the investigation of the influence of a single factor, the forward simulation is based on the following assumptions. First, the material of each part in the model is homogeneous, especially for the surrounding rock, and the influence of the joint and groundwater is not considered. Second, the intrinsic properties of the medium are determined, and will not be changed by the influence of the electromagnetic field.

### 3.1. Establishment of Model

**Figure 3:**
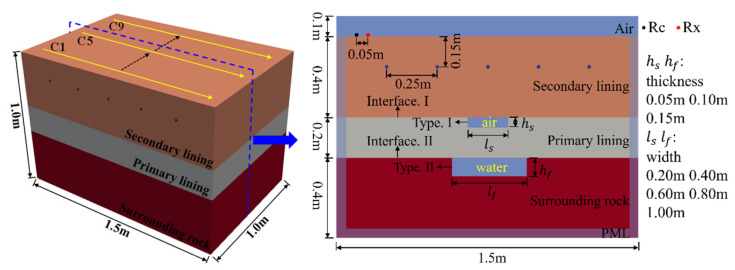
Proposed forward model. The figure illustrates the size and position of the components in the model, including the number of measuring lines, and the range of size variation of voids.

Generally, the tunnel lining consists of two parts: the primary lining and the secondary lining. The void generally occurs at the interface between the primary lining and the secondary lining or the surrounding rock, and most of the voids are air-filled. However, owing to the influence of groundwater, the void may also be water-filled, especially at the interface between the primary lining and the surrounding rock. Because the dielectric constant of concrete is very different from that of air and water, GPR can be used to detect voids in the lining. Support structures for tunnels can be divided into two types: plain concrete support without steel suitable for good geological conditions and reinforced concrete support used to deal with large deformation of the surrounding rock and the seismic load. Based on these conditions, a forward model was established to simulate the process of tunnel lining detection using GPR, as shown in Figure 3. The model was divided into four layers, namely, air, secondary lining, primary lining, and surrounding rock. Furthermore, the secondary lining was divided into two types: plain concrete (Support A) and reinforced concrete (Support B). The air-filled void was set at the interface between the secondary lining and the primary lining (interface Ⅰ), and the water-filled void was set at the interface between the primary lining and the surrounding rock (interface II). The effects of different void widths ($l_{s}$: the width of air-filled void; $l_{f}$: the width of water-filled void) and thicknesses ($h_{s}$: the thicknesses of air-filled void; $h_{f}$: the thicknesses of water-filled void) were considered in each type of void, and only one type of void was considered in each working condition. Nine lines were set up on the lining surface to study the response of voids in the detection with the 3D GPR. The distance between the measuring lines is 0.1 m. Among them, C1 and C9 do not pass through the void, C2 and C8 pass through the edge of the void, C3~C7 pass through the void, and C5 passes through the center of the void. It should be noted that the perfect matching layer (PML) was set in the model, and an area of 0.1 m width was reserved at the boundary of the model to generate a sufficient number of cells to absorb the electromagnetic waves transmitted to the boundary of the model. According to Giannopoulos [31], this technique can effectively reduce the error of simulation results caused by the reflection of electromagnetic waves at the boundary of the model. The discrete mesh size of the model was determined using Equations (5) and (6).

### 3.2. Parameters Determination

The determination of the model parameters is divided into two steps. First, the electrical parameters of the medium were selected; second, the detection parameters of the GPR were determined, considering the medium parameters.

First, selecting the electrical parameters. Forward modeling requires four electrical parameters of the medium: relative permittivity, conductivity, relative permeability, and magnetic loss. These four parameters control the response of the void. The conductivity, relative permeability, and magnetic loss are generally considered to be the loss and attenuation of the electromagnetic wave, and the influence on the electromagnetic wave velocity is considered only at low frequencies. Therefore, for the 400–900 MHz high-frequency GPR commonly used in lining detection, only the influence of the relative permittivity was considered. The electrical parameters of air and steel are built in the gprMax, and the electrical parameters of the primary support and secondary lining were valued based on [32]; the electrical parameters of the surrounding rock were determined based on moist granite, given by the Engineering Geology Manual [33]. The parameters were further selected through forward modeling of the lining detection.

Second, determining the detection parameters. The energy of electromagnetic wave will attenuate rapidly when it propagates in the medium, and the attenuation amplitude is positively related to the frequency. It is necessary to choose the appropriate frequency for tasks with different detection depths. Field cases and experiments show that GPR with 500 MHz can detect the target with a required buried depth while maintaining a good resolution [34,35]. Therefore, in this study, the frequency of 500 MHz was selected as the signal source. Because the model is a multi-layer structure, with reference to the method given by Xiao et al. [36], the equivalent relative permittivity of the lining and surrounding rock was estimated, as shown in Equation (7).

(7) $$\varepsilon_{dx}={\left(\sum_{i=1}^{n}\frac{h_{i}}{H}\sqrt{\varepsilon_{i}}\right)}^{2}$$

where $\varepsilon_{dx}$ is the equivalent relative dielectric constant of the multilayer media; $\varepsilon_{i}$ is the relative dielectric constant of each layer; H is the buried depth of the target, 0.75 m; and $h_{i}$ is the propagation distance of electromagnetic wave in each layer.

It is necessary to set the continuous acquisition time of each electromagnetic wave, that is, the time window. The time window should be set according to the maximum detection depth and the electromagnetic wave velocity in the medium, as shown in Equation (8). At the same time, it should be slightly larger than the two-way travel time of electromagnetic waves. Referring to the suggestion of “Technical regulation for geological radar detection of highway tunnel” [37], the coefficient k=1.3 was determined.

(8) $$W=\frac{k\times 2H}{\nu }=\frac{k\times 2H*\sqrt{\varepsilon_{dx}}}{\nu }$$

where W is the time window; v is the propagation velocity of electromagnetic waves in vacuum, 0.3 m/ns; and k is the expansion coefficient, 1.3.

The distance between the measuring points in the forward simulation should ensure that the response of the medium does not overlap in space. According to Nyquist’s law, the distance between measuring points should be one-quarter of the neutron wavelength of the medium, and it should also be less than the minimum size of the detection target, as shown in Equation (9).

(9) $$d=\frac{75}{f*\sqrt{\varepsilon_{\max}}}<d_{\min}$$

where d is the distance between the measuring points and f is the frequency of the GPR.

The parameters of the forward model were preliminarily selected using the above-mentioned methods. To further verify the rationality of the used parameters, the simulation results were compared with the measured results. 

**Figure 4:**
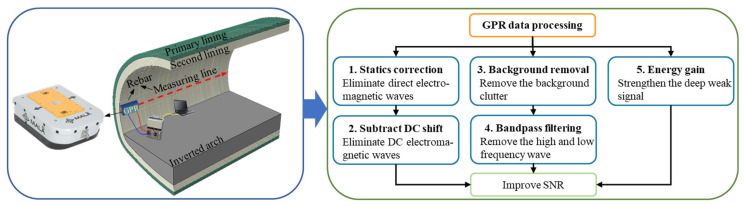
MALA GPR system with 500 MHz for tunnel side wall detection. The SNR was improved by five steps of data progress.

**Figure 5:**
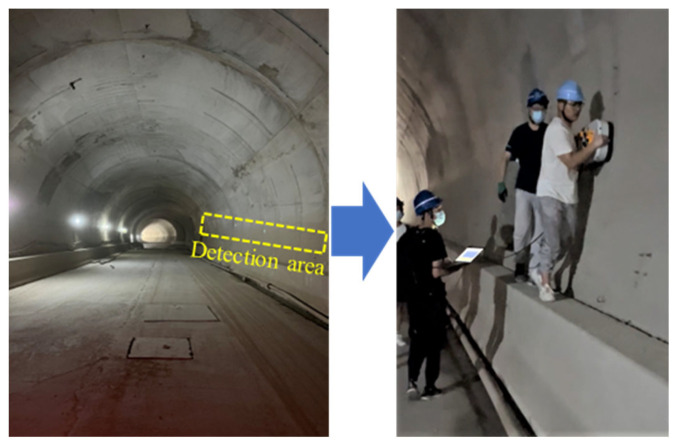
On-site data acquisition of GPR in a mountain tunnel.

**Figure 6:**
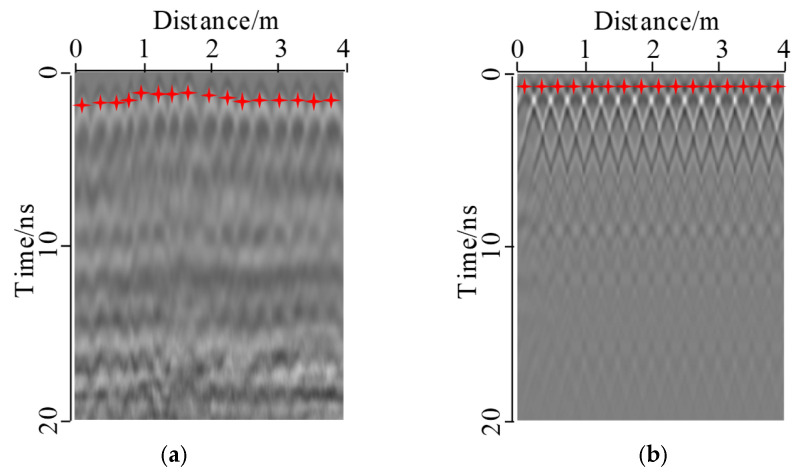
Comparison between simulation and measurement: (**a**) Measured image, (**b**) Forward image. One steel bar was set in the range of four meters, and obvious diffraction waves were observed, which shows that the establishment of the forward model is reasonable.

MALA GPR system was used to obtain the measured detection data, as shown in Figure 4. The on-site detection uses a distance measuring wheel to ensure the sampling interval is 0.01 m, the frequency of the antenna is 500 MHz, and the measuring line is located at the tunnel side wall. In order to remove the interference signal and improve the signal-to-noise ratio (SNR), the GPR data are processed as follows: static correction, subtract DC shift, bandpass filtering, background removal, and energy gain. The main points of data processing are shown in Figure 4, and the principle can be found in the manual of Reflexw [38]. The on-site detection of GPR in a mountain tunnel is shown in Figure 5. The resulting image is shown in Figure 6. By comparison, it was found that the steel bar in the lining could be clearly observed with a strong ringing signal response, but the measured signal was weaker than the simulated result through the forward model. This difference was due to the fact that the measured lining had a higher moisture content, which blocked the propagation of electromagnetic waves and reduced the signal strength. However, the influence of water was not considered in the forward model. In engineering practice, concrete and surrounding rock are not homogeneous, and the difference in local electrical characteristics will also lead to further attenuation of the signal. On the other hand, compared with the regular arrangement of steel bars in the simulation results, the distribution of steel bars in the measured images was uneven, which was mainly due to the extrusion of concrete during construction. The simulation results were close to the measured images, so it can be considered that the parameters of the model were appropriate. The used forward simulation parameters are listed in Table 1.

## 4. Analysis of Forward Simulation

### 4.1. Non-Void Lining

**Figure 7:**
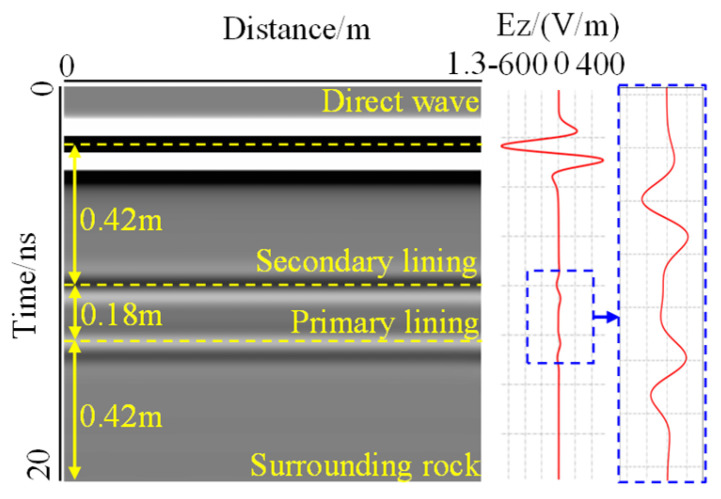
Forward image of support A. The figure contains two clear interfaces, electromagnetic waves fluctuated at the interfaces. The thickness of each layer is close to that in the forward model.

**Figure 8:**
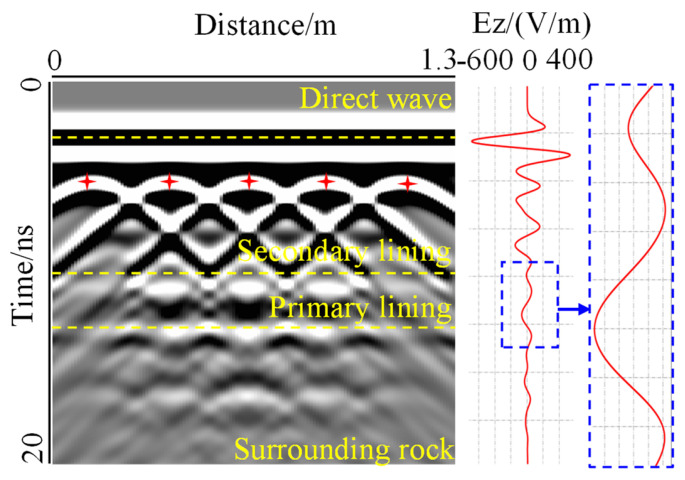
Forward image of support B. The steel bars caused the strong reflection and diffraction of electromagnetic waves, which covered the reflected signals from interface Ⅰ and Ⅱ.

The response of the non-void lining of two types of support (Support A and Support B) and electric field intensity was obtained by forward simulation, as shown in Figure 7 and Figure 8. For these two cases, there was a strong response at the interface between the air and the secondary lining, which was due to the large change in relative permittivity at the interface, where the electromagnetic wave was reflected. Some of the electromagnetic wave was received directly by the receiver as a direct wave without entering the medium, and the two-way travel time of the direct wave was 3 ns. The direct wave is a type of interference signal for GPR detection, and its two-way travel time cannot reflect the actual depth.

For Support A, as shown in Figure 7, two interfaces can be clearly seen in the forward image, with two-way travel times of 10 ns and 12.5 ns, respectively. Electromagnetic waves fluctuated at the interface. The thickness of the secondary lining and surrounding rock obtained by the forward simulation was 0.42 m, the thickness of the primary support was 0.18 m, and the error was 2%, which showed that the forward simulation had good accuracy. This again proved the rationality of the model setting. The layering characteristics can be reflected through forward simulation results according to the time-history parameters.

For Support B, as shown in Figure 8, it was found that the electromagnetic wave was strongly reflected and diffracted in the steel bar. The response characteristic of the steel bar was a hyperbola with an opening downward, and the position at the top of the hyperbola is the position of the steel bar. It can clearly be seen that the distance between the steel bars was 0.25 m, which is consistent with the model setting. Under the influence of diffraction waves, the tails of hyperbolas superimposed each other, creating strong interference, resulting in signal fluctuations in the initial branch and surrounding rock that cannot be clearly presented. However, the time required for electromagnetic waves to pass through each layer was not affected by diffraction, so the images of reinforced concrete can be stratified according to the two-way travel time of electromagnetic waves obtained by the forward modeling of Support A.

### 4.2. Influence of Void Width

For 500 MHz antennas, the horizontal resolution can be calculated by Equation (10). The horizontal resolution at interface Ⅰ and interface Ⅱ is 0.4 m and 0.6 m, respectively.

(10) $$R_{h}=\sqrt{\lambda H}$$

where $R_{h}$ is the horizontal resolution and λ is the wavelength of the 500 MHz antenna, 0.6 m.

**Figure 9:**
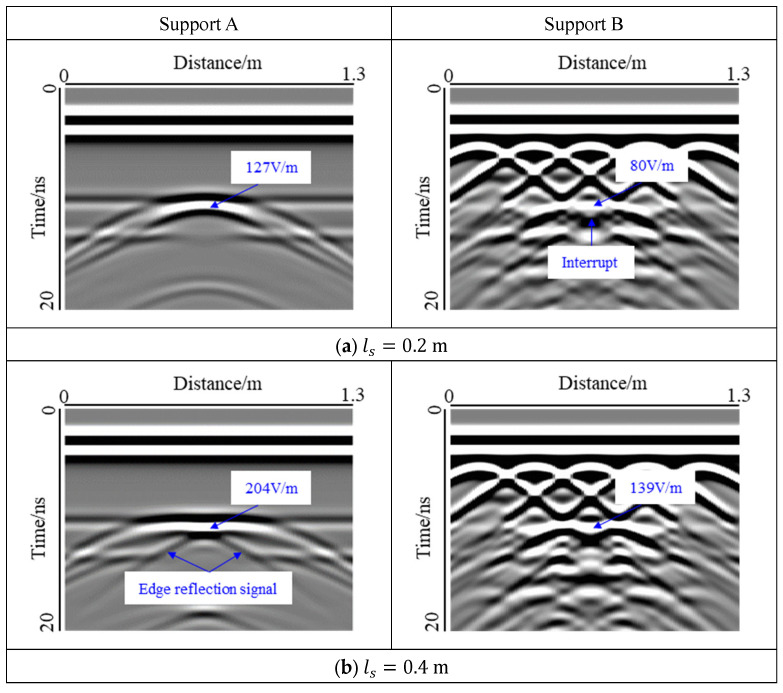

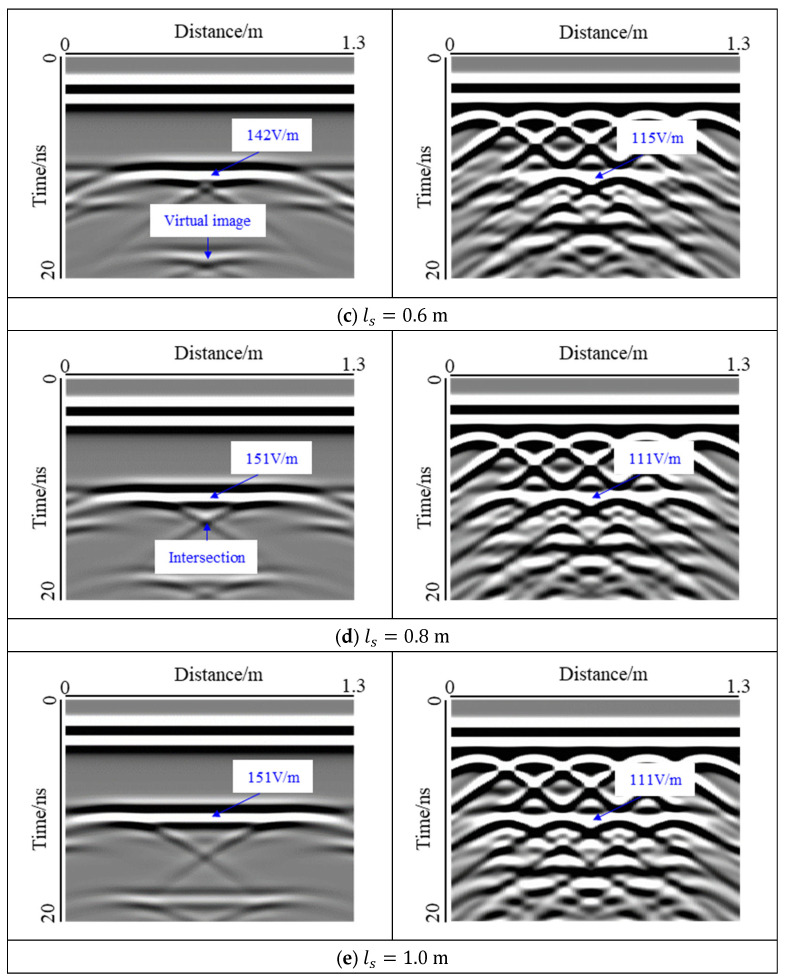
Influence of an air-filled void width at interface I. The increase in the void width caused the change in the response mode, and the interface Ⅱ was gradually covered by the void response.

The voids with a thickness of 0.1 m were selected, and the effect of the width on the void response was studied. The void at interface I, which is an air-filled void, was studied as shown in Figure 9. At the same time, the electric field strength of the void center is marked.

For Support A, two interfaces were observed in the forward simulation, when the void width was small. With an increase in the void width, the response of the interface was masked by the void response and could no longer be identified. The response of the void top was the strongest. With an increase in the void width, the response gradually penetrated the model, the reflection at the edge of the void became more dispersed, and the shape of the void response changed from hyperbolic to bowl-shaped. When $l_{s}\;$= 0.2 m, the width of the void was smaller than the horizontal resolution of GPR, and the reflected signal at the edge of the void was covered by the response from the top of the void. When 0.4 m < $\;l_{s}\;$ < 0.6 m, the width of the cavity reached the horizontal resolution of GPR, and the signal at the edge of the void could not be recognized. The reflected signal at the edge began to appear and approached the center of the void, until at 0.8 m <$\;l_{s}\;$< 1.0 m, the two reflected signals showed the mode of a cross. Thus, it can be seen that the response mode of the void is closely related to the width; most notably, the response area at the top of the void and the form of the reflected signal at the edge. This result was also observed by Luo et al. [39] through forward simulation and experiments. The reason for this change is the different ratios of void spread to the radar footprint. At the same time, the electromagnetic wave was reflected many times in the void, resulting in a virtual image similar to that of the void response in the lower part of the model, which cannot be used as the basis for evaluating the void. For Support B, the diffraction wave of the steel bar interfered with the response of the void. As a result, the reflection signal at the edge of the void and the virtual image of the lower part of the model were suppressed and became invisible. However, the response of the top of the void was still clearly visible and gradually expanded with an increase in the width of the void. When $l_{s}\;$ = 0.2 m, the response of the top of the void was interrupted by the diffraction wave.

According to the statistics of the electric field strength at the center position of the void top, it was found that for the two support modes, the electric field strength increased at first and then decreased with the width, and had the maximum value when $l_{s}\;$= 0.4 m, but no longer changed when $l_{s}\;$> 0.8 m. This change was mainly affected by the reflection response at the void edge. When $l_{s}\;$= 0.4 m, the two reflections were concentrated in the center of the void, which increased the response intensity. With an increase in the width, the two reflections gradually separated. At that time, the strengthening effect on the response of the void center region was no longer obvious. At the same void width, the electric field intensity of the void center in Support B was smaller than that in Support A owing to the interference of the diffracted waves.

**Figure 10:**
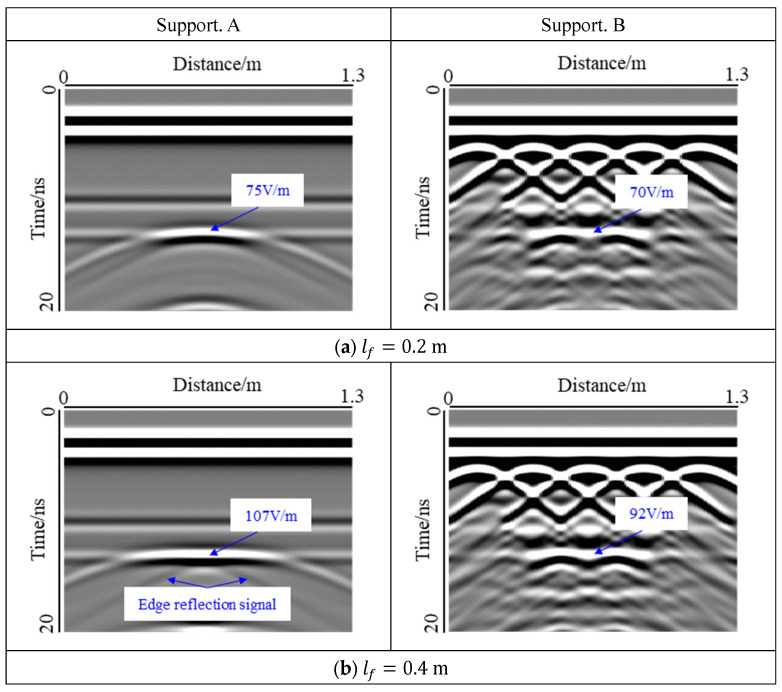

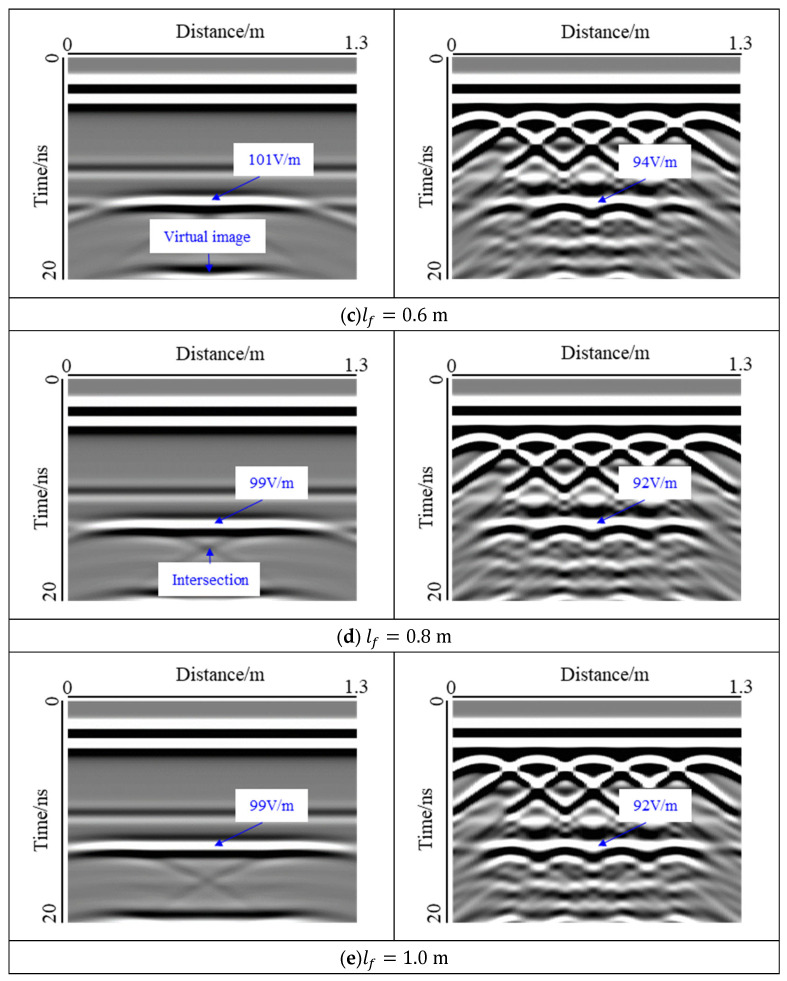
Influence of the water-filled void width at interface Ⅱ. Because the void was far away from the steel bar, the interference of the diffracted wave was smaller, and the void response could be observed more clearly.

The void at interface Ⅱ, which is a water-filled void, was studied as shown in Figure 10.

For Support A, interface Ⅰ could be clearly seen in the forward results, whereas interface Ⅱ was disturbed by the void response. The change in void response with width was similar to that of interface I. The response mode of the void changed from hyperbola to bowl-shape. When $l_{f}\;$ = 0.4 m, the reflected signal of the void edge was detected, and the width of the void did not reach the horizontal resolution of GPR at the interface Ⅱ, which was caused by the low-frequency oscillation of electromagnetic wave in water. When $l_{f}\;$ = 1.0 m, the void response ran through the model, the response of interface Ⅱ was completely covered, and the response mode changed to a strip-shape. The top of the void had a strong response, and with an increase in the void width, the reflection signal of the void edge gradually moved closer to the center until the shape of the cross appeared. The virtual image caused by multiple reflections also appeared at the bottom of the image, but it was not fully presented because of the limitation of the time window and the PML boundary of the model. For Support B, compared with the void at interface I, because the void was far away from the steel bar, the interference of the diffracted wave was smaller, and the void response could be shown more clearly. Owing to the influence of the refraction response and the multiple reflection response at the corner of the void, the interference of the steel bar diffraction wave was no longer obvious in the area below the void.

According to the statistics of the electric field strength at the center position of the void top, it was found that the electric field strength was weaker than that at interface I, because the electromagnetic wave was further dissipated at the primary lining. In addition, the water in the void significantly blocked the propagation of electromagnetic waves. This change law was consistent with the observed result at Interface I and had a maximum value at $l_{f}\;$ = 0.4 m.

### 4.3. Influence of Void Thickness

For 500 MHz antennas, the vertical resolution can be calculated by Equation (11). The vertical resolution at interface Ⅰ and interface Ⅱ is 0.3 m. The thickness of the void (0.05 m, 0.10 m, and 0.15 m) considered in this study does not reach the vertical resolution of GPR.

(11) $$R_{v}=\frac{\lambda }{2}$$

where $R_{v}$ is the horizontal resolution.

**Figure 11:**
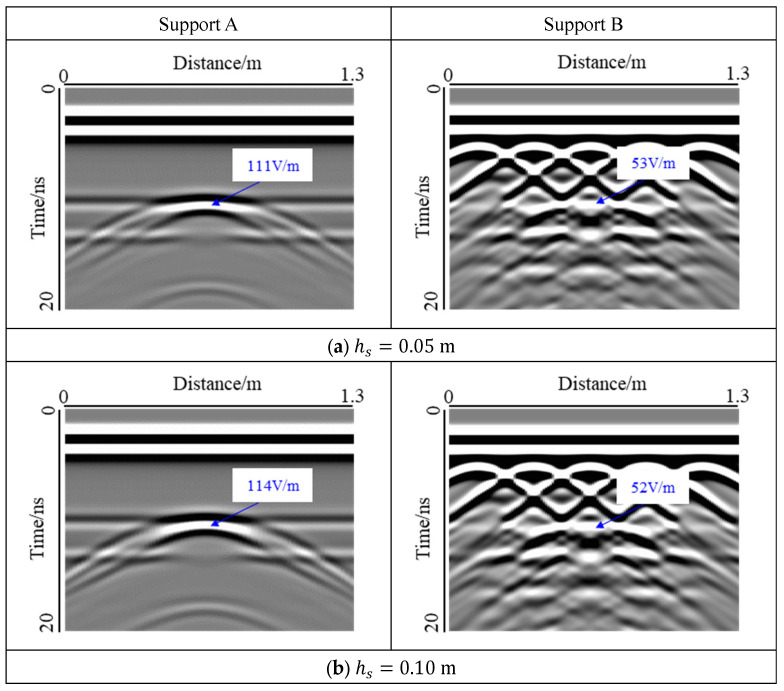

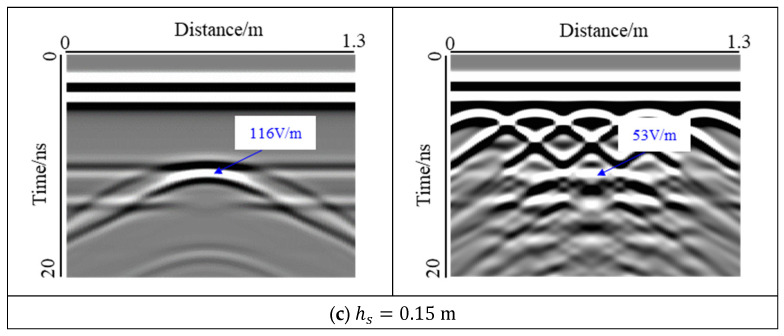
Influence of air-filled void thickness at interface I. The figure shows that the thickness has little influence on the void response and electric field intensity; therefore, it is difficult to judge the void thickness by 2D GPR.

A void with a width of 0.2 m was selected, and the effect of thickness on the void response was studied, as shown in Figure 11.

For Support A, the change in thickness mainly affected the extension region of the void response, and the reflection response at the void edge was more concentrated, which was difficult to distinguish manually. For Support B, the influence of the diffracted wave of the steel bar was even smaller, and the void response images of the three types of thicknesses were almost the same. At the same time, according to the statistics of the electric field strength at the center of the void, it was found that the effect of the void thickness could be almost ignored. Therefore, based on the detection results, it was concluded that the layering of the lining, the positioning of the steel bar, the location of the void position, depth, and width could be obtained, but it was difficult to determine the void thickness when applying 2D GPR in lining detection. This is because for the common void size behind the tunnel lining, the thickness is less than the vertical resolution of GPR, so it is difficult to identify the upper and lower interface of the void. Increasing the GPR frequency can improve the resolution, but the void at interface II may not be detected because the energy of high-frequency electromagnetic waves decays faster in the medium. Therefore, in tunnel detection, it is necessary to select the appropriate antenna frequency according to the detection task.

### 4.4. Influence of Water-Filled and Air-Filled Voids

**Figure 12:**
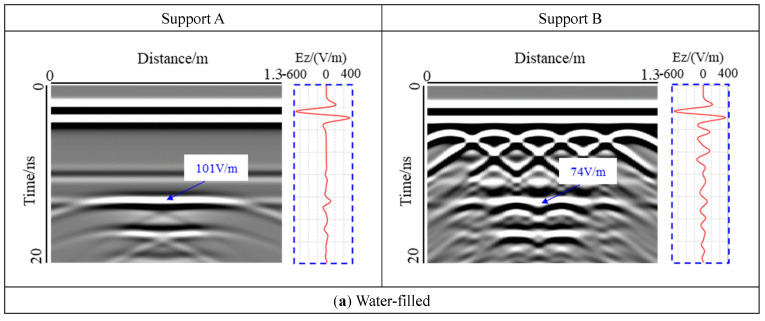

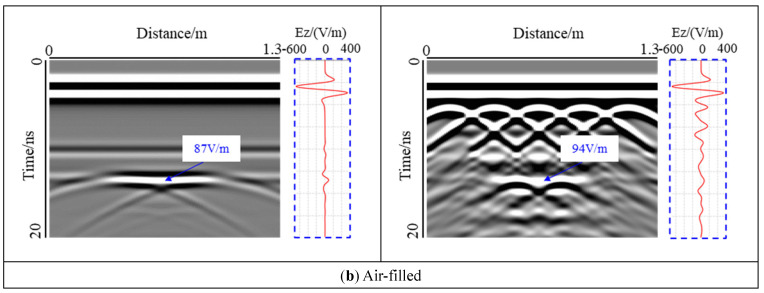
The influence of water-filled and air-filled. Compared with the air-filled void, water filling increases the response range of the void and produces a virtual image.

The effects of air-filled and water-filled voids on the response were analyzed by selecting the void at interface Ⅱ, with $l_{f}\;$= 0.6 m, $h_{f}\;$= 0.05 m, as shown in Figure 12.

It was found that there was a virtual image in the lower part of the model for the water-filled void, while there was no virtual image in the lower part of the air-filled void, mainly because the relative dielectric constant of water is much larger than that of air. Water greatly hinders the propagation of electromagnetic waves, resulting in electromagnetic waves in the void having a strong amplitude of low-frequency oscillations for many times, forming a virtual image. In addition, the response range of water-filled voids was larger than that of expandable voids. In the central part of the model, the electromagnetic wave was emitted from the excitation source and passed through the secondary lining, the primary lining, the void, and the surrounding rock. Because the relative permittivity of each layer was different, the phase of the electromagnetic wave changed at the interface. For the air-filled void, the relative permittivity of air was smaller than that of the primary lining, the reflection coefficient of the electromagnetic wave at interface I was positive, and the electromagnetic wave curve was not reversed at this position. For the water-filled void, the reflection coefficient was negative, and the electromagnetic wave curve was reversed. When the support structure was plain concrete in Support A, for the water-filled void, the electromagnetic wave had a positive phase at interface II, but it had a negative phase in the air-filled void. For reinforced concrete in Support B, the electromagnetic wave response inside the void showed a positive phase owing to the interference of the steel bar diffraction wave. Therefore, for the lining of the plain concrete support, the void internal filling or expansion can be evaluated by the phase change of the electromagnetic wave at the interface.

### 4.5. Statistics of Void Response Area

**Figure 13:**
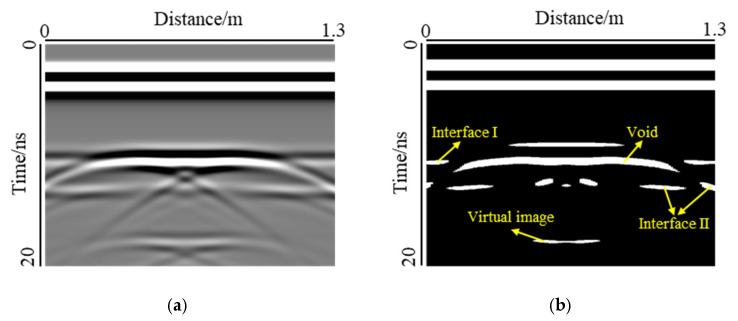
Binarization treatment: (**a**) Forward result (**b**) Binary image. In a binary image, the white area represents a stronger GPR response.

From the above analysis, it can be seen that the change in void shape, especially the change in width, has an obvious influence on the mode of void response. In this section, the threshold method was used to binarize the forward simulation results, and the area of the void response was calculated. Because of the large interference caused by the diffracted wave of the steel bar in Support B, the area of void response cannot be calculated accurately, so only the influence of void thickness and void width on the response area was considered under the condition of Support A. A typical binarization effect is illustrated in Figure 13.

**Figure 14:**
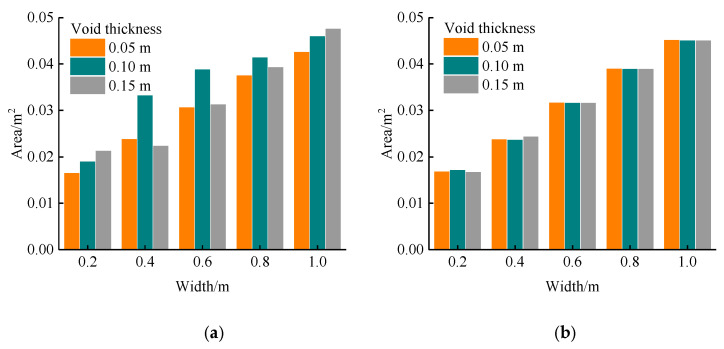
Void response area: (**a**) Interface I, (**b**) Interface Ⅱ. This figure shows the effect of width and thickness on the void response area. The area of the void response increased significantly with an increase in the void width, but the effect of the void thickness on the response area was not regular.

In the binarization image, the void response can be observed as a long strip, while the two interfaces are no longer complete. The response of interface I is distributed at the edge of the image, while the response of interface II is distributed not only at the edge of the image, but also in the lower part of the void response. The virtual image caused by multiple reflections of the electromagnetic waves can also be displayed in the binarized image. The binarization image is in good agreement with the void response of the forward modeling result. The void response area was calculated, as shown in Figure 14.

**Figure 15:**
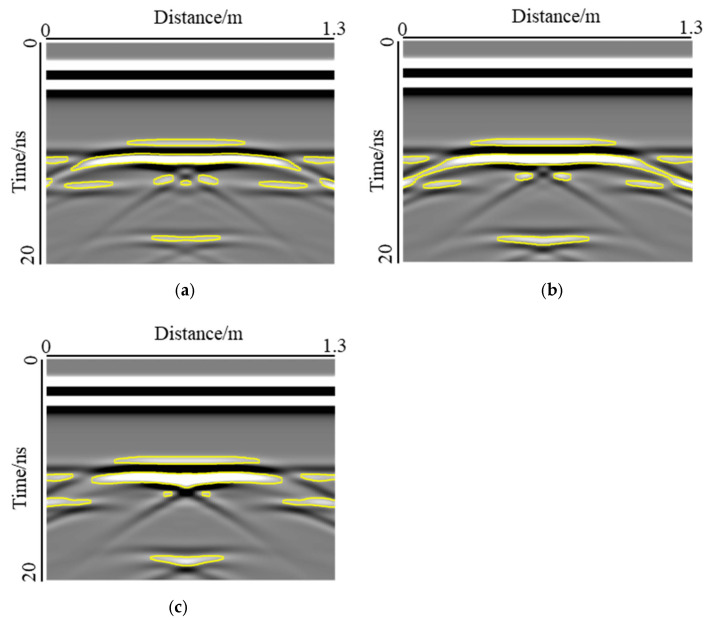
Effect of thickness on the extension area of the void response: (**a**) Bowl-shaped, $h_{s}=0.05\;m;$ (**b**) Bowl-shaped, $h_{s}=0.10\;m;$ (**c**) Strip-shaped, $h_{s}=0.15\;m$. This figure shows the different response of the void when the thickness changed.

The statistical results for the voids at interface I are shown in Figure 14a. Under the three types of void thickness, the response area was positively correlated with the width, and the area of the void response increased significantly with an increase in the void width. The effect of the void thickness on the response area was not regular. When the width was 0.2 m, the area of the void response increased gradually with an increase in thickness. Because the void size was smaller, the edge of the void did not produce an obvious diffraction signal, and the response mode of the void was hyperbolic. The change in thickness mainly affected the extension area of the void response, as shown in Figure 9. When the width was in the range of 0.4–0.8 m, the void response mode changed from hyperbolic to bowl-shaped. When the thickness was 0.05 m and 0.10 m, the two ends of the void response extended downward, and the change in the thickness led to the difference in the extension area between the two ends of the void response, as shown in Figure 15a,b. However, when the thickness was 0.15 m, the void response was strip-shaped, and the two ends of the response no longer extended downward, as shown in Figure 15c. When the width was 1.0 m, the void responses of the three thicknesses were all through the model. The void responses of the thicknesses of 0.05 m and 0.10 m were bowl-shaped, and the void responses of the thicknesses of 0.15 m were strip-shaped. In this case, the area of the void response was positively correlated with the thickness.

From the statistical results for the void at interface II as shown in Figure 14b, it can be observed that the area of the void response was positively correlated with the width. In addition, the area of the void response was basically the same for the three types of void thickness. This was because the void at interface II was closer to the bottom of the model, and more electromagnetic waves were absorbed by the PML boundary, resulting in less clutter. When the void thickness was greater than 0.4 m, the void anomaly response was strip-shaped, and the banded length varied with the void length.

**Figure 16:**
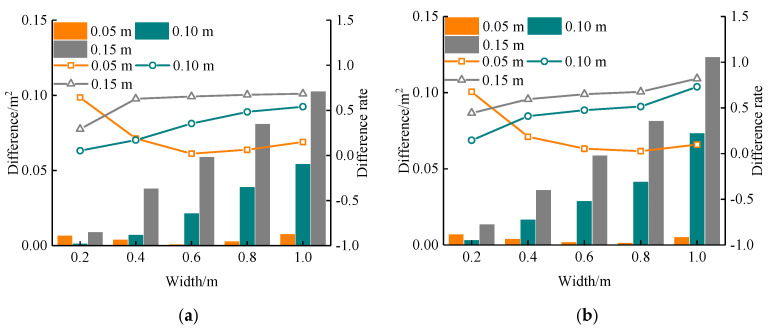
Difference between calculated area and actual area: (**a**) Interface Ⅰ, (**b**) Interface Ⅱ. This figure shows the effect of width and thickness on the difference between calculated area and actual area.

The response area and actual area were compared, as shown in Figure 16. It can be observed that for the void at the two interfaces, the difference between the response area and the real area increased with the change in the void width. When the thickness was 0.05 m, the difference rate first decreased and then increased, but the difference rate increased continuously when the thickness was 0.10 m and 0.15 m. Therefore, there is a significant error in predicting the void area by using GPR, especially when the void thickness is large. The error will increase rapidly with the increase in void width. However, when the void thickness is small, within a certain range of void width, the prediction of the void area by GPR detection image can have a better result (width less than 0.4 m, thickness less than 0.1 m). This is because, for the common void size behind the tunnel lining, the thickness is less than the vertical resolution of GPR. GPR cannot distinguish the upper and lower boundaries of the void, and the response model of the void is similar. The change in thickness mainly affects the extension region and electric field strength of the void response, and the average thickness of the cavitation response is similar. Under the same binarization threshold, the statistical response area has little difference, but the actual void area is increasing, resulting in an increase in error. Therefore, the use of GPR to estimate the void area is suitable for the case of small void thickness.

### 4.6. Three-Dimensional forward Simulation of Void

From the previous analysis, it can be seen that the change in width and thickness will lead to a change in the void response mode, but it is difficult to evaluate the relevant morphological parameters by GPR. With the development of GPR technology, 3D GPR has been used in lining detection, and it can obtain more information about the tunnel lining. In this section, a three-dimensional forward model was established by selecting the void at interface I with a width of 0.6 m and thickness of 0.1 m (Figure 3). The 3D data volume of the void was obtained by setting nine measuring lines, and the void features were obtained by slicing the data volume along the measuring line in the horizontal direction.

**Figure 17:**
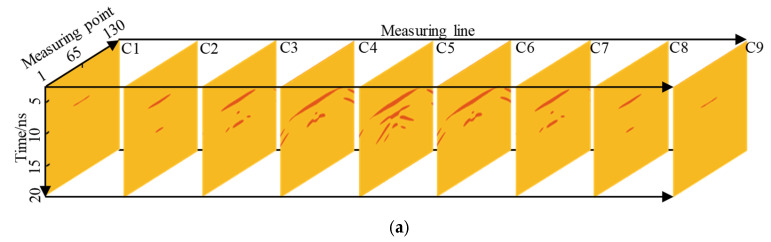

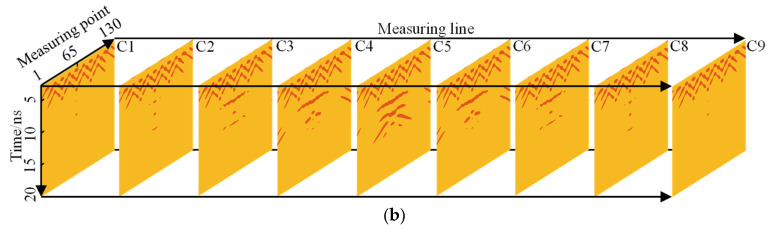
Slices along the measuring line: (**a**) Support A, (**b**) Support B. This figure illustrates that the void response increases as the measuring line approaches the center of the void.

Figure 17 shows the slice along the measuring line. For Support A, as shown in Figure 17a, it can be observed that as the measuring line approaches the center of the void, the void response increased gradually, reaching a maximum at C5, and the reflection at the edge of the void was also gradually enhanced. Therefore, when the GPR is used to detect the lining, the measuring line should be allowed to pass through the void central area as much as possible to obtain a stronger void response. C1 did not pass through the void, but there was also a slight response, which may lead to a miscalculation of the void position. For Support B, as shown in Figure 17b, the law is similar to that of Support A, and the void response was the strongest at the center of the void. The intensity of the same line response disturbed by the steel bar diffraction wave was less than that of Support A.

**Figure 18:**
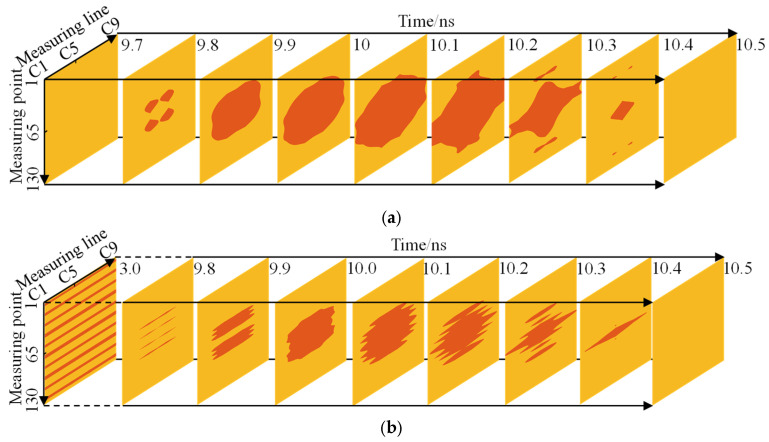
Slices along the horizontal direction: (**a**) Support A, (**b**) Support B. This figure illustrates that the response of the center of the cavity is the strongest.

Figure 18 shows the slice along the horizontal direction. For Support A, as shown in Figure 18a, it can be found that with an increase in time, the void response appeared at 9.8 ns, the response area was scattered, and the position corresponded to the four corners of the void. Subsequently, the response area gradually became large and connected with each other, which was close to the oval shape. The response area was the largest during 10.1 ns, and the slice passed through the horizontal center of the void. As the slice was farther away from the void, the response area gradually shrank to the center of the void until it disappeared completely. For Support B, as shown in Figure 18b, the time depth of the first slice is 3 ns, which was the area affected by the diffracted wave of the steel bar. It can be observed that there were 10 strip responses along the measuring point because the electromagnetic wave produced diffracted waves on both sides of the steel bar, and the position of the steel bar was between the two responses. The void response was stripped at first, and with the increase in time depth, the response area increased gradually, reaching a maximum of 10.1 ns, and the response area was smaller than that of Support A. Then, the response showed a strip, and the region began to decrease until it finally disappeared. This change was mainly affected by the ringing signal of the steel bar.

From the above analysis, it can be seen that when using 3D GPR to detect the lining, the images of plain concrete support and reinforced concrete support are quite different, but the slices along the measuring line and horizontal slices have the strongest response when they pass through the void center plane, so the central position of the void can be determined according to this law.

## 5. Conclusions and Discussions

In this study, a forward model based on FDTD was proposed to study the GPR response of the void behind tunnel lining with different conditions. It was assumed that the material in the model was homogeneous and its intrinsic properties were determined. The influence of different variables on the void response characteristics was comprehensively analyzed to provide a reference for the evaluation of voids in actual engineering situations. The conclusions are summarized as follows.

(1) The change in void width affects the mode of void response, which can be divided into hyperbolic, bowl-shaped, and strip-shaped. The void response area is positively correlated with the width of the void.

(2) The area of void response was calculated and compared with the area set in the model. It was found that the estimation of the void area by GPR was more accurate for the case of small void size (width less than 0.4 m, thickness less than 0.1 m), and the error increased with an increase in the size of the void.

(3) The influence of void thickness on GPR response is very small, especially, when there is a steel bar in the lining. Due to the interference of diffraction waves, it is difficult to judge the void thickness by 2D GPR.

(4) The measuring line that passes through the center of the void has the strongest response; this result can be used as a reference to accurately determine the center location of the void using 3D GPR.

This study discussed the factors affecting the void response mode. In the analysis, all voids were supposed to be smooth and continuous, while in practical engineering, voids are irregular and discontinuous, which will have an impact on the void response mode. Further research is required to consider the void shape. In addition, due to the construction error and the instability of maintenance conditions, the hydration reaction of concrete is not complete, which will lead to a false setting and initial defects in concrete. Therefore, concrete is not an ideal uniform medium, which will also interfere with the propagation of electromagnetic waves. Therefore, a random medium should be used to simulate concrete.

## Figures and Tables

**Table 1:** Model parameters.

Grid Size	∆x (m)	∆y (m)	∆z (m)	
	0.005	0.005	0.005	
GPR	Frequency (MHz)	Excitation	Spatial step (m)	Time window (ns)
	500	Ricker	0.01	20
Medium	Relative Permittivity	Conductivity (mS/m)	Equivalent relative permittivity	Equivalent velocity (m/ns)
Second lining	7.5	0.005	6.19	0.12
Primary lining	5.0	0.005		
Rock	8.0	0.001		
Water	81.0	0.03		

## Data Availability

Not applicable.
